# A model for preemptive maintenance of medical linear accelerators—predictive maintenance

**DOI:** 10.1186/s13014-016-0602-1

**Published:** 2016-03-10

**Authors:** Charles M. Able, Alan H. Baydush, Callistus Nguyen, Jacob Gersh, Alois Ndlovu, Igor Rebo, Jeremy Booth, Mario Perez, Benjamin Sintay, Michael T. Munley

**Affiliations:** Department of Radiation Oncology, Wake Forest School of Medicine, Medical Center Boulevard, Winston-Salem, NC 27157 USA; Department of Radiation Oncology, Florida Cancer Specialist, 8763 River Crossing Boulevard, Florida, USA; Gibbs Cancer Center and Research Institute, Spartanburg Regional Medical Center, Greer, SC USA; John Theuer Cancer Center, Hackensack University Medical Center, Hackensack, USA; North Sydney Cancer Center, Royal North Shore Hospital, Sydney, Australia; Cone Health Cancer Center, 501 N. Elam Avenue, Greensboro, NC 27403 USA

**Keywords:** Quality control, Quality assurance, Predictive maintenance, Statistical process control, Radiation therapy, Linear accelerator

## Abstract

**Background:**

Unscheduled accelerator downtime can negatively impact the quality of life of patients during their struggle against cancer. Currently digital data accumulated in the accelerator system is not being exploited in a systematic manner to assist in more efficient deployment of service engineering resources. The purpose of this study is to develop an effective process for detecting unexpected deviations in accelerator system operating parameters and/or performance that predicts component failure or system dysfunction and allows maintenance to be performed prior to the actuation of interlocks.

**Methods:**

The proposed predictive maintenance (PdM) model is as follows: 1) deliver a daily quality assurance (QA) treatment; 2) automatically transfer and interrogate the resulting log files; 3) once baselines are established, subject daily operating and performance values to statistical process control (SPC) analysis; 4) determine if any alarms have been triggered; and 5) alert facility and system service engineers. A robust volumetric modulated arc QA treatment is delivered to establish mean operating values and perform continuous sampling and monitoring using SPC methodology. Chart limits are calculated using a hybrid technique that includes the use of the standard SPC 3σ limits and an empirical factor based on the parameter/system specification.

**Results:**

There are 7 accelerators currently under active surveillance. Currently 45 parameters plus each MLC leaf (120) are analyzed using Individual and Moving Range (I/MR) charts. The initial warning and alarm rule is as follows: warning (2 out of 3 consecutive values ≥ 2σ _hybrid_) and alarm (2 out of 3 consecutive values or 3 out of 5 consecutive values ≥ 3σ _hybrid_). A customized graphical user interface provides a means to review the SPC charts for each parameter and a visual color code to alert the reviewer of parameter status. Forty-five synthetic errors/changes were introduced to test the effectiveness of our initial chart limits. Forty-three of the forty-five errors (95.6 %) were detected in either the I or MR chart for each of the subsystems monitored.

**Conclusion:**

Our PdM model shows promise in providing a means for reducing unscheduled downtime. Long term monitoring will be required to establish the effectiveness of the model.

## Background

Medical electron linear accelerators have continued to evolve since their development in the late 1950’s at Stanford University [1]. Modern medical accelerators are complex digital devices producing multiple photon and electron beams. These accelerators are comprised of a number of auxiliary systems that facilitate the treatment of cancer using an expanding range of clinical approaches. Accelerator design has interwoven hardware and software such that the opportunity to control components and systems with increasing precision has also increased the ability to monitor system operation. While the manufacturing and design of medical electron accelerators has improved the reliability and consistency of operation, system dysfunction and failure still occur.

Currently, digital data accumulated in the accelerator system is not being exploited in a systematic manner. This untapped resource could be used to assist in identifying operational deviations that can improve efficient deployment of service engineering resources resulting in fewer interruptions to service. Linear accelerator interlocks ensure that the operation of the system is discontinued when parameters exceed the limits of a system’s operating specifications. Component failure or dysfunction requires immediate repair and service engineering on site. The result is unscheduled machine downtime and disruption of clinical services. Our previous work has determined that often failure is preceded by a gradual and measurable deviation of the component’s normal operational parameters [2–7].

The purpose of this study was to develop an effective process for detecting unexpected deviations in accelerator system operating parameters and/or performance that predicts component failure or system dysfunction. This way maintenance can be performed prior to the actuation of interlocks. Performance characteristics critical to the delivery of high quality dynamic treatment (Volumetric Modulated Arc Therapy, Intensity Modulated Radiation Therapy, Sterotactic Body Radiation Therapy, Gated delivery) are: (a) gantry speed and positional fidelity, (b) MLC leaf speed and positional fidelity as a function of gantry position, and (c) beam uniformity (steering) as a function of gantry position. We hypothesize that accelerator systems under optimal circumstances operate with random variation that can be modeled by a continuous probability density function. By establishing the mean operating values using Statistical Process Control (SPC) methodology [8–14] and continuously sampling these values we can detect unexpected deviations that predict component failure or system dysfunction. The use of performance data within a systematic, integrated SPC framework can be deployed as a Predictive Maintenance (PdM) program for medical linear accelerators.

The proposed predictive maintenance (PdM) model is as follows: 1) deliver a daily QA treatment; 2) automatically transfer and interrogate the resulting log files; 3) once baselines are established, subject daily operating and performance values to statistical process control (SPC) analysis; 4) determine if any alarms have been triggered; and 5) alert facility and system service engineers. A key component of this work was the development of software modules to automate the interrogation of trajectory log files, perform the SPC evaluation, and display the results in a graphical dashboard interface.

## Methods and materials

The work reported here is the result of a project involving a data collection partnership (DCP) of five facilities monitoring seven digital accelerators (3 - True Beam and 4 - True Beam STX radiotherapy systems, Varian Medical Systems, Inc). Partners were asked to deliver a robust VMAT treatment (Snooker Cue, Van Esch et al. [15]) each day. The log files resulting from the treatment were transferred, decoded, analyzed, regrouped and subjected to SPC analysis. The field service reports for each accelerator were submitted and tracked in tandem with the SPC analysis. The project has been conducted in the run-to-failure format, meaning that no active service intervention is initiated solely as a result of data analysis. Each facility maintained autonomy in determining when and what level of accelerator maintenance would be performed.

### Daily QA treatment delivery and file transfer

Trajectory and text log files are written for each treatment delivery and are accumulated on the accelerator server. The daily VMAT QA treatment delivery was designed to assess the interplay between gantry angle, MLC position and dose delivery in a single treatment [15]. Characteristics of Snooker Cue delivery that were of particular interest were the dose delivery at narrow angular sectors that provided maximal gantry acceleration and deceleration, and the delayed displacement of the MLC gap from one position to the other which enforced maximum leaf speed before coming to an abrupt halt at the moment of delivery. The four subarc fields provided by Dr. Van Esch’s team were integrated into a single delivery using the automated delivery feature of the accelerator.

Each DCP facility was provided a cloud storage account and the ability to sync a local folder. Automated daily log file transfer allows for daily analysis and review.

### File decoding, data analysis and regrouping

All computer code is written in MatLab (The MathWorks, Inc, Natick, MA, USA). The trajectory and text log files are decoded by separate functions. The text log is written at the start of the delivery and contains a single snapshot of 45 data values. There is no processing of these data. The trajectory logs contain parameter data taken every 20 msec during the delivery. Each trajectory file is decoded and a total of 131 axis positions were chosen to be recorded (collimator jaw position, gantry angle, each MLC, etc.). These raw data are processed and axis positions are extracted at critical points during the delivery. Where axis velocity is evaluated, it is determined by positional change over time. The focus of our analysis is the accuracy, reproducibility and fidelity of each axis. A reference positional trace of the gantry and each MLC is used as a motion baseline for cross correlation (CC) analysis. The trajectory logs of 494 parameters were analyzed, 482 of which were MLC related. A total of 525 operational or performance parameters are monitored (35 text log and 490 trajectory log) using SPC analysis.

### SPC analytical formulism and evaluation guidelines

In this project the use of the I/MR chart is the overall optimal choice since each value is identified with a specific period of time—daily. Since it is impractical to deliver the QA treatment multiple times each day, the logical subgroup size for single daily delivery is *n* = 1. While I/MR charts are the most sensitive in identifying changes in parameters, our methods for calculating the chart limits and chart analysis rules will determine how effective we can predict when maintenance intervention is necessary. Our experience has shown that the use of traditional SPC chart limits (±3 standard deviation (±3 *σ*) from the grand mean) can result in an unacceptable rate of false positive signals [5, 6]. Using information on system specifications from the manufacturer, consulting the literature for recent studies on quality control of complex treatment delivery [16, 17] (IMRT, VMAT, SBRT, etc.) and white papers on quality assurance of linear accelerators [18, 19], we introduce a hybrid approach to calculating the control chart limits that includes a factor (*S*_*p*_) that fractionally increases the limits based on the operational parameter specification and/or performance criteria $\left(\pm 3\ {\sigma}_{{}_{\mathrm{hybrid}}}\right).$

The magnitude of empirical factor *S*_*p*_ is dependent upon the informational source:

- System specification: up to 10 % of the operating range or specification
- Published quality control requirements: up to 1 % of the quality control absolute value/range or,
- Values developed based on controlled experiments carried out by the predictive maintenance team on multiple accelerator systems.

Individual grand mean (*Ī*), moving range (MR) and grand mean (I/MR) chart values are determined as follows:

1 $$ \overline{I}=\frac{\left[{\displaystyle \sum }{I}_{t_{{}_{t=1\ .\ .\ .\ T}}}\right]}{T} $$

2 $$ M{R}_t = \left|{I}_t - {I}_{t+1}\right| $$

3 $$ \overline{MR}=\left[{\left({\displaystyle \sum }M{R}_t\right)}_{t=1\ .\ .\ .\ T-1}\right]/\left(T-1\right) $$

where *T* = 20 (for initial use but the value of T may be altered at a later date by the user via the dashboard interface) and I_t_ is the individual value of the component. Control limits are then calculated.

For individual upper and lower control limits are defined as:

4 $$ \overline{I}\pm \left[\left({E}_2\overline{MR}\right)+{S}_p\right] $$

5 $$ 3\ {\sigma}_{{}_{\left(\mathrm{I}\right)\mathrm{hybrid}}} = \left[\left({E}_2\overline{MR}\right)+{S}_p\right] $$

For moving range control limits:

6 $$ UCL=\left[{D}_4\overline{MR}\right]+{S}_p $$

7 $$ LCL={D}_3\overline{MR}=0 $$

8 $$ 3\ {\sigma}_{{}_{\left(\mathrm{R}\right)\mathrm{hybrid}}} = \left[\left({D}_4\overline{MR}\right)+{S}_p\right] $$

where factors (E_2_, D_3_, D_4_) are constants that are determined by sample size [14]. The effect of using our hybrid approach is to increase the variance and kurtosis of the parameter probability density function.

A SPC data repository consisting of a multidimensional matrix is created for each accelerator along with several reference files specific to its baseline performance. Individual chart warnings and alarms are determined by:

- Alarms: 2 of 3 or 3 of 5 consecutive data points exceeding the upper or lower control limits (≥ ±3σ _(I)hybrid_);
- Warnings: 2 of 3 or 3 of 5 consecutive data points exceeding ±2 ${\sigma}_{{}_{\left(\mathrm{I}\right)\mathrm{hybrid}}}$ from the grand mean.

Moving Range chart warnings and alarms are determined by:

- Alarms: 3 of 5 consecutive data points exceeding the upper control limit (≥3σ _(R)hybrid_);
- Warnings: 3 of 5 consecutive data points exceeding +2 ${\sigma}_{{}_{\left(\mathrm{R}\right)\mathrm{hybrid}}}$ from the mean.

### Dashboard interface development

The development of a dashboard interface to display and evaluate the results of the SPC charts is an integral part of this project. The dashboard is modeled after traditional SPC charts used in manufacturing [9] that include the control charts, a horizontal frequency distribution of data samples, statistical summary of control chart parameters and other data, as well as insertion and display of user comments for each parameter. Additional features include user determined chart limit revisions, zoom in/out, and report generation. The MATLAB programming environment was used to develop the predictive maintenance dashboard (PMD).

### Synthetic errors introduced

Synthetic errors were introduced to determine the initial effectiveness of the I/MR charts for detecting relevant changes in operating parameters. Table 1 list the parameters and errors introduced. These errors were introduced as a singular shift/deviation in parameter value on consecutive days.

**Table 1:** Synthetic deviations introduced - parameter, magnitude, and detection result

No.	PARAMETER	ERROR DESCRIPTION	ERROR LEVEL	DETECTION
1	PFN High Voltage Power Supply Current (A)	5 % based on operating ranges	1.05	YES
2	PFN Actual Voltage (KV)	5 % based on operating ranges	1.05	YES
3	RF Driver Voltage (V)	1STD	0.5	YES
4	RF Forward Power (W)	1STD	0.11	YES
5	AFC Error (V)	1STD	0.03	YES
6	Gun Current (A)	No change—add	0	N/A
7	Gun High Voltage (V)	1STD	559	YES
8	Gun Grid Voltage (V)	1STD	7.94	YES
9	Gun Filament Step Voltage (V)	Half of minor fault change	0.1	YES
10	Gun Filament Voltage (V)	Half of minor fault change	0.1	YES
11	Bend Magnet Current (A)	1 % change	1.01	YES
12	Bend Magnet Voltage (V)	5 % change	1.05	YES
13	Accelerator Solenoid Current (A)	1STD	1	YES
14	Klystron Solenoid Current (A)	.100 A change	0.1	YES
15	Radial Symmetry (%)	0.5 % added	0.5	YES
16	Transverse Symmetry (%)	0.5 % added	0.5	YES
17	Target Current (nC)	1STD	11	YES
18	Buncher Radial Current (A)	1STD	0.13	YES
19	Buncher Transverse Current (A)	1STD	0.1	YES
20	Angle Radial Current (A)	Shift/add 0.2 A	0.2	YES
21	Angle Transverse Current (A)	Shift/add 0.2 A	0.2	YES
22	*Position Radial Current (A)*	Shift/add 0.2 A	0.2	*NO*
23	Position Transverse Current (A)	Shift/add 0.2 A	0.2	YES
24	Trim: (A)	1STD	0.055	YES
25	Accelerator Vacion Current (uA)	Add .1 % of Vac2 fault value	0.007	YES
26	Positive 5 V dc	Shift/add 0.1 V	0.1	YES
27	*Positive 24 V dc*	Shift/add 0.1 V	0.1	*NO*
28	Analog Negative 5 V dc	Shift/add 0.1 V	0.1	YES
29	Analog Positive 5 V dc	Shift/add 0.1 V	0.1	YES
30	Negative 12 V dc	Shift/add 0.1 V	0.1	YES
31	Positive 3 V dc	Shift/add 0.1 V	0.1	YES
32	Node Power Supply Voltage (V)	Shift/add 0.1 V	0.1	YES
33	Water Level	No change—add	0	N/A
34	Internal Water Supply Temperature (deg C)	1 degree change	1	YES
35	Gas Pressure (PSI)	1 psi	1	YES
36	Y1	Add 0.2 cm	0.2	YES
37	Y2	Add 0.2 cm	0.2	YES
38	X1	Add 0.2 cm	0.2	YES
39	*X*2	Add 0.2 cm	0.2	YES
40	Carriage A	Add 0.2 cm	0.2	YES
41	Carriage B	Add 0.2 cm	0.2	YES
42	Gantry—Speed 1, Speed 2	Add 0.2 deg/sec	0.2	YES
43	Gantry—cross-correlation max value	Shift 10 snapshots	0.2 deg	YES
44	*Gantry—location of cross-correlation max value*	Shift 10 snapshots	0.2 deg	*NO*
45	MLC Bank A (60 leaves)—each leaf: Speed 1, Speed 2	Add 0.1 cm/sec	0.1	YES
46	MLC Bank B (60 leaves)—each leaf: Speed 1, Speed 2	Add 0.1 cm/sec	0.1	YES
47	*MLC Bank A (60 leaves)—each leaf: cross-correlation max value*	Shift 2 snapshots	1 mm	*NO*
48	MLC Bank A (60 leaves)—each leaf: location of cross-correlation max value	Shift 2 snapshots	1 mm	YES
49	*MLC Bank B (60 leaves)—each leaf: cross-correlation max value*	Shift 2 snapshots	1 mm	*NO*
50	MLC Bank B (60 leaves)—each leaf: location of cross-correlation max value	Shift 2 snapshots	1 mm	YES

### Text log parameters

The system parameters monitored using text files include RF generation, electron gun control, energy control, beam uniformity control, DC voltage generation, and cooling systems. Synthetic errors/changes were introduced in all but two of the parameters (gun current and water level). The magnitude of the synthetic errors/changes were based on:

- the value of 1 standard deviation from the mean operating parameter of 483 clinically deployed accelerator systems
- a small fraction (≤5 %) of the operating range (provided by manufacturer)
- a fraction of the minor fault deviation (provided by manufacturer).

### Trajectory log parameters

The accelerator system axes (127) monitored includes collimator jaw position, MLC carriages, gantry angle, and each MLC (treatment couch axes are excluded). There are 490 parameters (482 MLC related) analyzed from the trajectory log files. The magnitude of the synthetic errors/changes was based on: TG-142 [18] and published analysis of VMAT delivery accuracy [16, 17].

## Results

### Dashboard interface development and layout

**Fig. 1:**
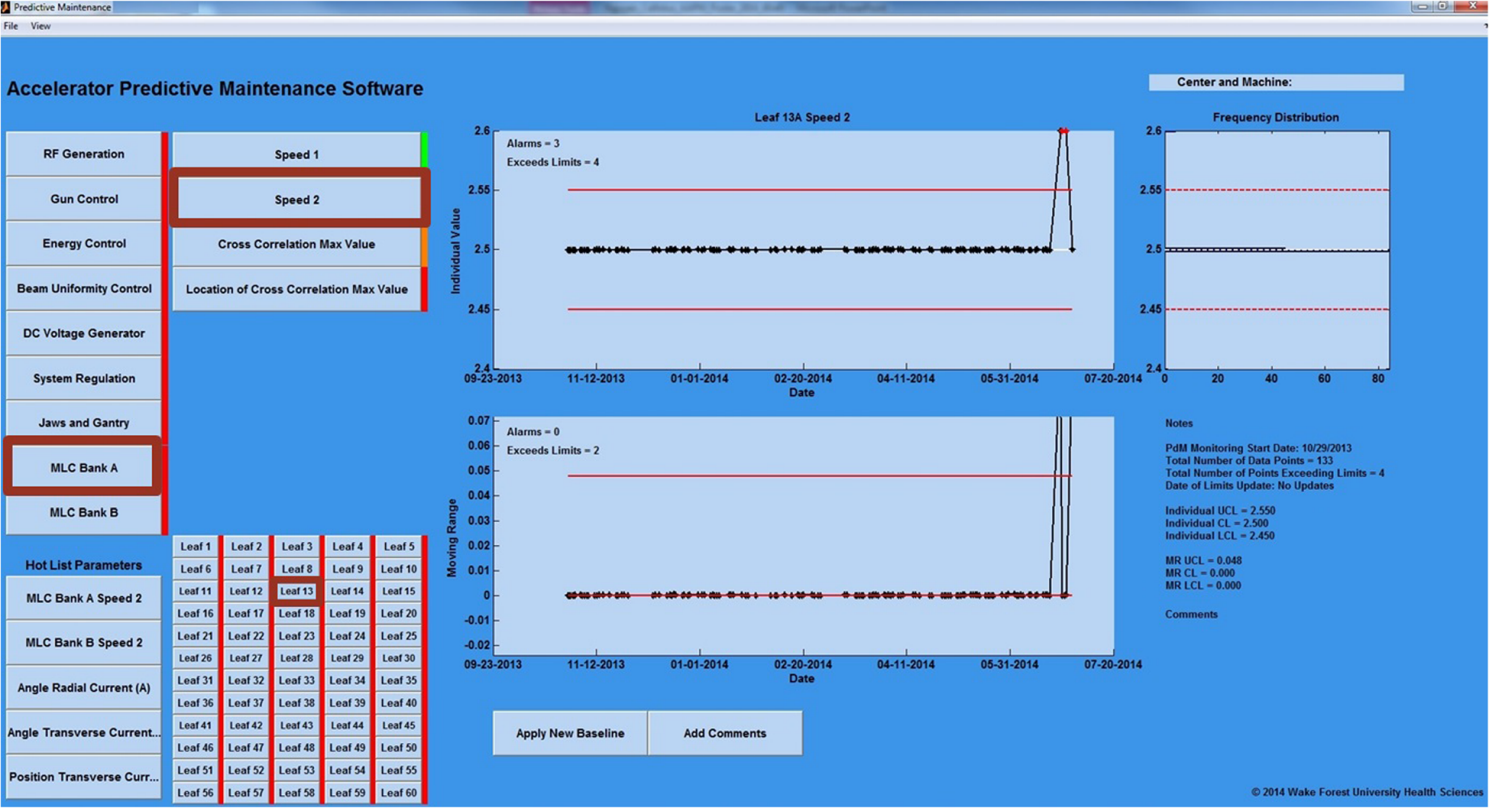
Linear accelerator predictive maintenance dashboard (PMD) illustrating the detection of a 1 mm/sec synthetic error. Subsequent monitoring shows the normal operation of the MLC continued at baseline level

**Fig. 2:**
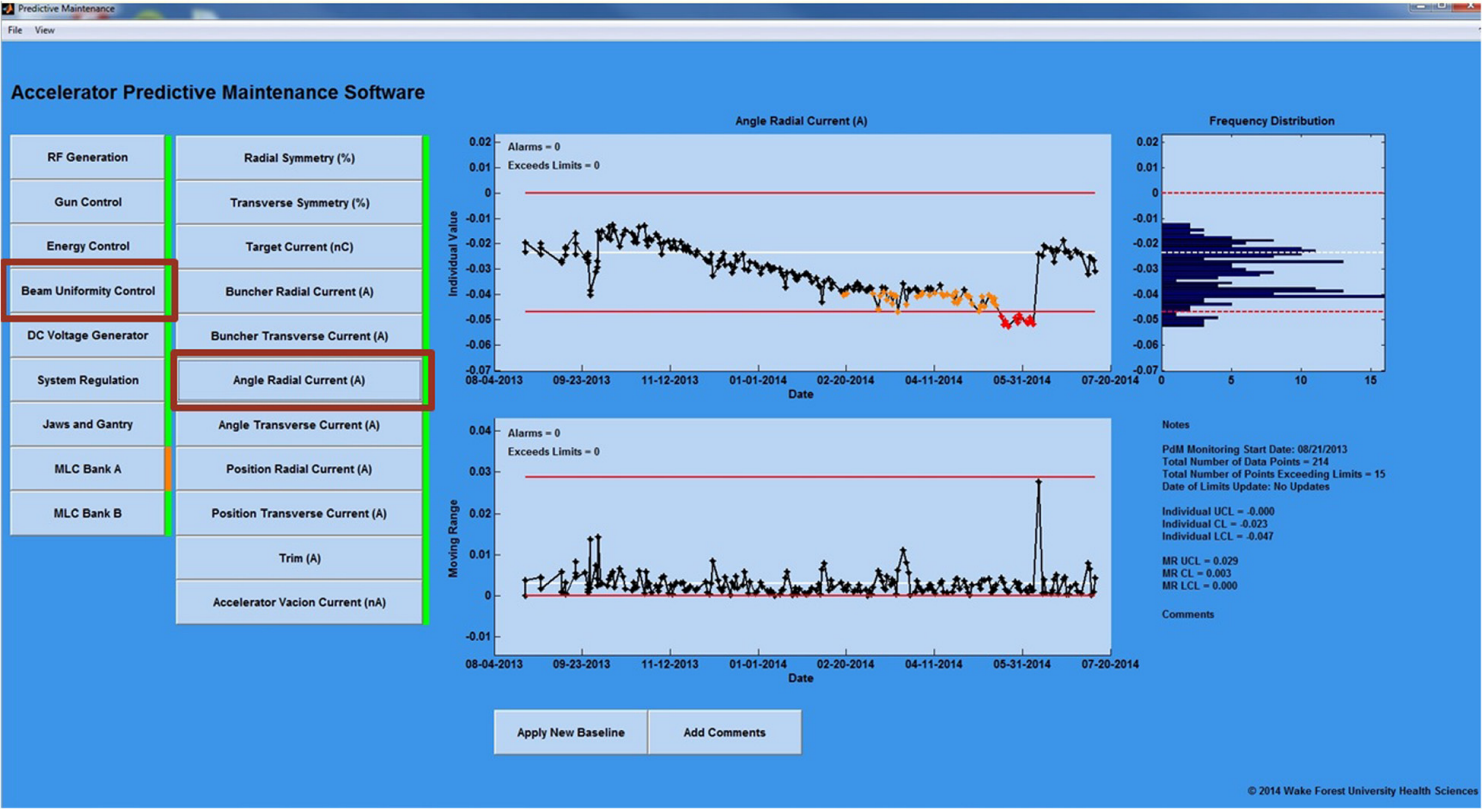
Linear accelerator predictive maintenance dashboard (PMD) illustrating a beam steering parameter (uniformity) change that was detected by the PdM process and confirmed via scanning water phantom. Continuous monitoring following service adjustments indicates beam steering was restored to baseline value

The PMD opens to a pane that prompts the user to identify the location of the data repository and associated reference files. Once accessed, a list of nine parameter groups (based on operational category) with a color-coded indicator of the status for rapid assessment of the group is shown. Red indicates an alarm state within the group, orange indicates a warning, and green indicates normal operation. Once a parameter group is toggled, a new pane opens and displays the parameters and their status lights. Parameter buttons activate display of the associated I/MR charts, frequency distribution of the I chart data, and a summary of important parameter information (Notes) and comments (Figs. 1 and 2). Alarm (red) and warning (orange) data points are indicated. In the I chart, the current number of alarms and points exceeding the limits are displayed in the upper left corner. These values are “current” for the last two work weeks (10 days) while a total tally is included in the “Notes” section. A “Hot List” of parameters in an alarm state is located just beneath the parameter groups for direct access to the analysis.

The PMD enables a user to change the range of data displayed and produce an output file or hardcopy of the chart under review. The user can update the control limits by toggling the “Apply New Baseline” button. The number of samples (T) used to determine the revised limits are user controlled as previously mentioned. Limit updates are warranted when a repair or adjustment has been made. The PMD prompts the user to enter the number of data points to be used in updating the control limits. An update of the control limits will change the limits for the last data point display and all future data. Relevant comments can be added by toggling the “Add Comments” button.

### Synthetic errors

The detection results are summarized in Table 1 alongside the parameter evaluated and the magnitude of the error introduced.

#### Text parameters

There were 33 subsystems or components in which synthetic errors were introduced. There were two (radial position steering coil, and positive 24 V DC) in which the errors did not exceed the limit of the I or MR chart. The I chart limit was exceeded for all of the remaining synthetic errors (93.9 %). The MR chart limit was exceeded in 29 (87.9 %) of the 31 parameters in which the I chart limit was exceeded.

#### Trajectory parameters

**Fig. 3:**
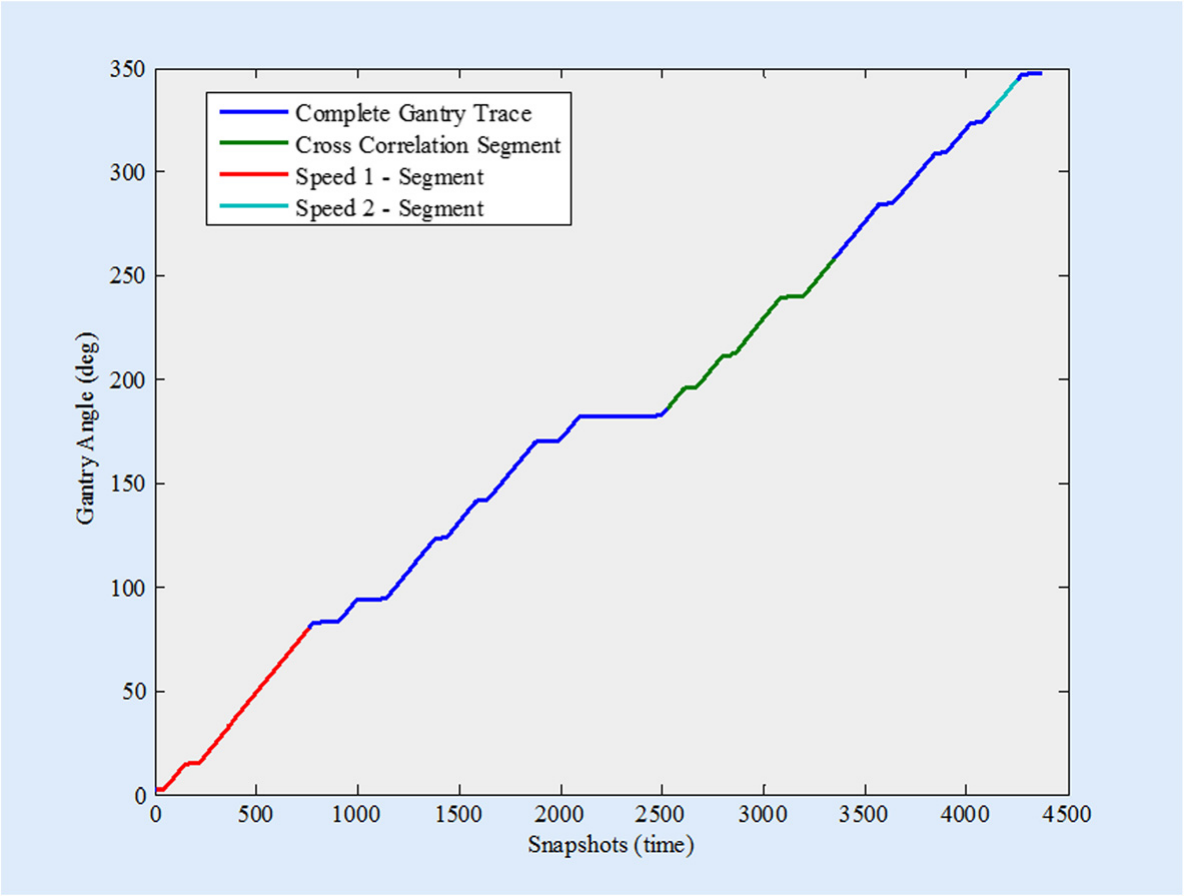
Graphical representation of the gantry position during the VMAT QA delivery. The Y-axis is gantry position in degrees and the X-axis is time in snapshots of 20 mSec. The segments of the delivery in which the speed is calculated are highlighted in red (snapshots 10–760) and light blue (snapshots 4120–4250). The segment used as the cross correlation baseline is show in green (snapshots 2525–3350)

**Fig. 4:**
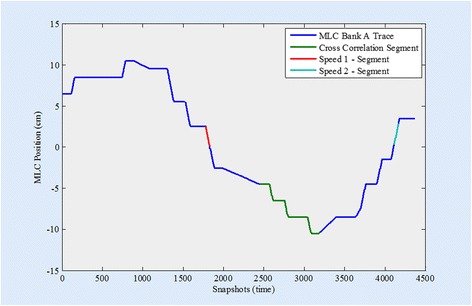
Graphical representation of the position of an MLC from bank A during the VMAT QA delivery. The Y-axis is MLC position in centimeters and the X-axis is time in snapshots of 20 mSec. The segments of the delivery in which the speed is calculated are highlighted in red (snapshots 1780–1830) and light blue (snapshots 4115–4165). The segment used as the cross correlation baseline is shown in green (snapshots 2450–3200)

There were 127 axes monitored using the trajectory log file data. The synthetic errors introduced were detected by at least one I/MR chart monitoring a related parameter. Velocity and cross-correlation tests were implemented at segments during the delivery that specifically challenged the system’s capability to perform the operation. The gantry and each MLC is monitored by two separate velocity I/MR charts at different points in the delivery. Positional fidelity of the gantry and each MLC is monitored by two different cross-correlation evaluation values: maximum value and location of maximum value. The non-random change in speed or position introduced by the synthetic error was detected in one of the speed or cross-correlation values for the gantry and all MLC leaves. The time and gantry position during delivery in which gantry speed is determined and cross-correlation evaluated is depicted in Fig. 3. The difference in position at the start and end of each speed segment is divided by the time to calculate gantry speed. MLC Bank A trace of position during delivery is depicted in Fig. 4.

#### System dysfunction detected and confirmed

Our analysis detected a warning condition on the radial angle steering coil current that suggested a deviation in the beam uniformity for the 6 MV photon beam on one of the accelerators (Fig. 2). The DCP facility physicist was notified to determine if there were any circumstances and/or supporting information that would explain the source of the change in this parameter. No service had been performed that could have resulted in the changes observed. A review of the daily flatness and symmetry measurements using a two point methodology indicated change of 1.0 % from baseline. According to the I chart and SPC analysis, the steering coil current entered a warning condition on March 6 which persisted until May 18 when it crossed into an alarm condition. It remained in alarm until the annual calibration on June 8. During the annual calibration the radial symmetry was found to differ by 0.8 % from the previous annual calibration confirming the I chart monitoring. The I chart demonstrates the radial angle steering coil current was returned to baseline following the adjustments made by service. The I chart detected the drift in the current value 51 days prior to it reaching an alarm level. In this case the PdM monitoring would have detected the change in the operating parameter early but did not alarm until almost reaching the TG-142 suggested 1 % constancy level.

## Discussion

Depending on the availability of engineering support, the impact of accelerator downtime on the facility and its patients can be vastly different. The ability to identify changes in the operation of subsystems prior to the actuation of an interlock that may require service can address this issue, particularly in regions of the world where the infrastructure of vendor engineering support may not be as strong as it is in the USA.

The design of the QA treatment delivered to assess the status of operation of the accelerator is critically important to the PdM model presented. The use of the Snooker Cue delivery allows us to explore the interplay between the gantry and MLC during VMAT. One of the limitations is that it was designed for delivery with a single photon energy (6 MV) and did not allow additional energies to be evaluated. Couch movements were not included in the delivery and therefore were not monitored. The Snooker Cue delivery was originally designed to be used with image evaluation of delivery performance. A custom designed synthetic treatment for PdM may be more effective in providing an overall evaluation of the accelerator system. Additionally, multiple deliveries of the QA treatment each day could be effective in uncovering operational drift during the course of the treatment day. The automation of several beams including static and VMAT including all photon energies that include movement of all axes may be more appropriate to the task of challenging accelerator operation to detect nonrandom changes in operation and performance.

Development of a hybrid approach to calculating chart limits for effective detection of nonrandom changes that are relevant to accelerator operation and performance was critical to our PdM monitoring. The inclusion of empirical factors based on historical system knowledge, engineering design, and performance quality standards is a practical approach to understanding how to customize the chart limits. In the future an analytical model to adjust chart limits would be more desirable as the volume of parameters being evaluated increases. Also, the tests employed to determine alarms needs to be expanded. We are therefore exploring the use of some alternative SPC chart tests commonly employed to help in identifying non-random changes.

## Conclusion

Our PdM model for preemptive detection of nonrandom changes shows promise in providing a means of reducing unscheduled downtime. We consider this work a first step in the process of detecting, interpreting, and presenting operational and performance data to predict impending accelerator subsystem dysfunction prior to the actuation of interlocks. Long term monitoring and correlation of service interventions will be required to establish the effectiveness of the model.
